# Other PHAC publications

**DOI:** 10.24095/hpcdp.45.10.05

**Published:** 2025-10

**Researchers from the Public Health Agency of Canada also contribute to work published in other journals and books. Look for the following articles published in 2025:**

Clay JM, Stockwell T, Golder S, Lawrence K, McCambridge J, Vishnevsky N, **Zuckermann A**, et al. The International Scientific Forum on Alcohol Research (ISFAR) critiques of alcohol research: promoting health benefits and downplaying harms. Addiction. 2025. https://doi.org/10.1111/add.70132

**Clayborne ZM, Capaldi CA, Mehra VM**. Associations between digital media use behaviours, screen time and positive mental health in youth: results from the 2019 Canadian Health Survey on Children and Youth. BMC Public Health. 2025;25(1):2303. https://doi.org/10.1186/s12889-025-22874-2

**Collins E, Edjoc R, Farrow A, Dharma C**, Georgiades S, **Holmes K, Orchard C, O’Donnell S, Palmeter S, […], Al-Jaishi A**. Prevalence of autism among adults in Canada: results from a simulation modelling study. BMJ Open. 2025;15(6):e089414. https://doi.org/10.1136/bmjopen-2024-089414

Lee EY, Park S, Kim YB, Liu H, Mistry P, Nguyen K, […] **Prince SA**, et al. Ambient environmental conditions and active outdoor play in the context of climate change: a systematic review and meta-synthesis. Environ Res. 2025;283:122146. https://doi.org/10.1016/j.envres.2025.122146

**Shields M, Tonmyr L, Pollock N**, Gonzalez A, **Hovdestad W, Tanaka M**, et al. Determinants of nonphysical intimate partner violence: a cross-sectional study with nationally representative data from Canada. Am J Epidemiol. 2025;194(6):1695-708. https://doi.org/10.1093/aje/kwae305

